# Vicarious Menstruation of a Novel Kind

**Published:** 1852-02

**Authors:** 


*Vicarious Menstruation of a Novel Kind.*—Dr. Lecointe, of Eu, in France, has published, in *L'Uuion Médicale*, a case of an extraordinary description, of of which we shall offer a brief outline. The subject is a servant girl, twenty-nine years of age, of apparently good health; she had never menstruated, and for the last seven years had experienced flushings and heat in the face, these symptoms recurring every four or five weeks. At these periods she likewise complained of severe lancinating pain in the right thigh, and sometimes leg and foot, the whole limb then becoming extremely tender to the touch.

Towards June, 1842, these phenomena increased in intensity, the patient became very weak, the abdomen felt tense, tympanitic, and tender, and she could no longer pass urine. Dr. Lecointe prescribed leeches to the hypogastrium, and prolonged hip-baths. The urine flowed a little; but at last the girl was persuaded to submit to the catheter, and a large quantity of dull and fetid urine was drawn off.

Now began a series of strange phenomena; the bladder, uterus, stomach, and rectum began to throw off what the patient called *balls;* these were pieces of membrane, or rather membranous casts, white, dense, and covered on one side with gelatinous matter. The vesical casts were somewhat large, as she was obliged to extract them with her fingers. On a former examination, the internal organs of generation could hardly be properly explored, as the hymen was unbroken, and rather tense, but the casts now came per vaginam, and the patient being obliged to dilate the parts herself, in order to give passage to the membranous formations, it was found on examination that the os was pervious, and the cervix of the normal size, though tilted backwards. Here the casts assumed a tubular shape.

The stomach became now very irritable, and a great abundance of glairy matter, mixed with pseudo-membranes, was thrown up. The vomiting was now and then of a purely sanguineous character, and in the coagula ejected an ascaris lumbricoides was noticed. The patient stated that she had likewise seen such parasites in the matters which had been expelled from the vagina. Diarrhea supervened a little time afterwards, and in the dejections the same pseudo-membranous products were observed. After a few weeks' respite, the symptoms recurred with renewed intensity; all the above-named organs secreted the same membranous products, but the uterus was evidently the most active. In one day Dr. Lecointe extracted ten casts from the vagina; they were rolled up, and exhibited now for the first time a red color. One of these presented on one side an infundibular shape, which made the medical attendant suspect that the membrane must have been formed in close vicinity to the Fallopian tube.

The ejection of the casts was always accompanied by much pain, the latter being sometimes so intense as to cause the patient, who was far from being pusillanimous, to roll about in the bed with agony. The sanguineous flux was now suddenly transferred to the ears; these organs discharged each about a tumblerful of blood; vomiting of the same fluid came on a few days afterwards, and the casts were again ejected from the stomach, intestines, bladder, and uterus. When these symptoms had ceased, a great improvement was noticed; the patient gradually became stronger, and from 1842 to the present time, the girl has experienced no uneasiness but dysuria every two or three months, except in July, 1843, when the most complete relapse occurred. The author does not say whether any amount of regular menstruation has appeared since the casts are no longer secreted. This is a great omission. The pseudo-membranous products were examined by M. Mialhe, and were found to be composed principally of albumen.—*Ibid.*

